# Stem cells: a new paradigm for disease modeling and developing therapies for age-related macular degeneration

**DOI:** 10.1186/1479-5876-11-53

**Published:** 2013-03-01

**Authors:** Heather Melville, Matthew Carpiniello, Kia Hollis, Andrew Staffaroni, Nady Golestaneh

**Affiliations:** 1 Georgetown University School of Medicine, 3900 Reservoir Rd, Washington, DC 20057, USA; 2 Department of Ophthalmology, Georgetown University, School of Medicine, 3900 Reservoir Rd, Washington, DC 20057, USA; 3 Department of Neurology, Georgetown University, School of Medicine, 3900 Reservoir Rd, Washington, DC 20057, USA; 4 Department of Biochemistry and Molecular & Cellular Biology, Georgetown University, School of Medicine, 3900 Reservoir Rd, Washington, DC 20057, USA

**Keywords:** Age-related macular degeneration, RPE, Stem cells

## Abstract

Age-related macular degeneration (AMD) is the leading cause of blindness in people over age 55 in the U.S. and the developed world. This condition leads to the progressive impairment of central visual acuity. There are significant limitations in the understanding of disease progression in AMD as well as a lack of effective methods of treatment. Lately, there has been considerable enthusiasm for application of stem cell biology for both disease modeling and therapeutic application. Human embryonic stem cells and induced pluripotent stem cells (iPSCs) have been used in cell culture assays and *in vivo* animal models. Recently a clinical trial was approved by FDA to investigate the safety and efficacy of the human embryonic stem cell-derived retinal pigment epithelium (RPE) transplantation in sub-retinal space of patients with dry AMD These studies suggest that stem cell research may provide both insight regarding disease development and progression, as well as direction for therapeutic innovation for the millions of patients afflicted with AMD.

## Introduction

Age-related macular degeneration (AMD) is a devastating neurodegenerative disease and leading cause of blindness in people over 55 years of age that affects a central nervous system tissue, the retinal pigmented epithelium (RPE) [1]. More than 11 million Americans over the age of 50 are affected by AMD, and with an aging population, this number will almost double by 2050 [2]. AMD is a multifactorial disease and its pathogenesis remains largely unknown, implying a complex interaction of genetic, environmental, metabolic and functional factors [3]. Clinically, AMD leads to the impairment of central visual acuity that is required for daily tasks such as reading, writing, driving, and recognizing faces, important for independent living. AMD occurs in two general forms, dry and wet. The dry form of AMD is characterized by polymorphic deposits called drusen that accumulate between the RPE and Bruch’s membrane [4]. The wet form is accompanied by choroidal neovascularization with subsequent formation of a disciform scar. Affected individuals may lose vision in both atrophic (dry) and the neovascular (wet) forms of AMD, however dry AMD is significantly more common, accounting for some 90% of total reported cases [5]. Dry AMD can transform into the wet form in approximately 10% of the patients, with devastating neovascularization-induced central vision loss [5]. There is currently no curative treatment for patients affected with AMD, with the best attempts seeking to forestall further degeneration at the retina [6]. Vitamin supplementation is recommended and is modestly beneficial for a small population of patients [7]. For the wet form of AMD anti-vascular endothelial growth factor (VEGF) therapy is applicable, however, the therapy is often administrated after significant damage has already been induced to the retina [8]. Consequently, the need for developing effective treatments to improve outcomes for patients with AMD is pressing [9]. Since the development of human embryonic stem cell lines in 1998 [10] and the advent of induced pluripotent stem cells [11,12] there has been enthusiasm in the scientific community for the potential utility of these cells in the understanding and treatment of AMD [13-18].

Stem cell biology can offer profound insight into the mechanisms of AMD [19] and can provide new approaches for autologous cell-based therapy in AMD as supported by the recently FDA approved clinical trial (NCT01344993). Generation of RPE derived from patient-specific induced pluripotent stem (iPS) cells may offer the ability to recapitulate the disease state and screen new therapeutics, improving upon the limited treatment strategies currently available to afflicted patients. This review will examine the breakthroughs and limitations of utilizing stem cells for disease modeling and therapeutic application in age-related macular degeneration.

## AMD: disease progression and etiology

Impairment in RPE functions in AMD induces loss of central vision at the macula as a result of photoreceptor degeneration [1]. RPE comprises a monolayer of pigmented cells with the apical membrane facing the light-sensitive outer segments of photoreceptors and the basolateral membrane facing the fenestrated capillaries of the choroid [20,21]. It plays many crucial roles in the retina including formation of blood/retina barrier by tight junctions, transportation of nutrients such as glucose or vitamin A from blood to the photoreceptors, conveyance of water from subretinal space to the blood, establishment of immune privilege of the eye, maintenance of ion composition in the subretinal space, light absorption, isomerization of retinal in the visual cycle, secretion of growth factors, and phagocytosis of the outer segments of the photoreceptors [22,23]. Due to their high metabolic activity, RPE cells are constantly subjected to oxidative stress and high levels of peroxidized lipid membranes [24]. Extended exposure to oxidative stress can disrupt RPE tight junctions, inducing the breakage of the blood barrier and producing abnormal membrane bleb structures [25,26]. Furthermore, impairment of RPE function in dry AMD can induce formation of abnormal extracellular deposits called drusen that accumulate between the RPE and Bruch’s membrane [4].Drusen, the clinical hallmark of AMD, consist of pathological extracellular deposits of degenerative material [4,27-31]. Drusen contain lipid and carbohydrate deposits, and have shown to include elements from both intracellular and extracellular sources. For example, integrins, lipoproteins, ubiquitin, inhibitor of metalloproteinase 3, advanced glycation end products, beta amyloid, fibronectin, and vitronectin have been identified in drusen [30,32-34]. Extracellular products include amyloid components, apolipoprotein E, factor X, immunoglobulin lambda chains, complement components, like the C1-q complex, late stage-activated complement components such as C5b-9 complex, and major histocompatibility complex (MHC) class II antigens [35]. Intracellular components are mainly derived from RPE and consist of cellular and basal lamina fragments, lipofuscin and melanin, organelles [36]. Some of the components of drusen are found in non-occular diseases. Similarities are found with amyloidosis, elastosis, and glomerular basement membrane disease [37]. Amyloid beta, a waste product that accumulates in the CNS with aging and Alzheimer’s Disease is a key component of drusen. Increased accumulation of amyloid beta with aging is found along Bruch’s membrane, blood vessels, and in the photoreceptor outer segment [38].

Genetic factors are now considered as reliable biomarkers to predict the risk of developing AMD, potential for disease severity and likelihood of progression [39]. Genetic studies of AMD determined by candidate gene approaches and genome wide association studies demonstrate the involvement of an inflammatory component [40]. Polymorphisms on chromosome 1 in complement factor H (*CFH*) [41], complement 2 (*C2*), complement factor B (*CFB*), complement 3 (*C3*), complement factor H-related gene (*CFHR1*) and complement factor I (*CFI*) are associated with increased risk of developing AMD [42-48]. Furthermore, polymorphisms on chromosome 10 in *ARMS2* (Age-related Maculopathy Susceptibility 2) [49] and the HTR1A serine peptidase 1 (*HTRA1*) genes predispose to wet AMD [49-52]. Polymorphisms in Apolipoprotein E (*APOE*), a component of drusen and a gene involved in lipid metabolism, appear to increase susceptibility to AMD [41,53,54]. Proteins with major roles in regulation of plasma lipids, such as hepatic triglyceride lipase (*HL*) and the cholesteryl ester transfer protein (*CETP*), as well as nearby markers of the inhibitor of metalloproteinase 3 (*TIMP3*) gene are also associated with an increased risk of AMD [40]. In addition, polymorphisms in *VEGFA*, a factor involved in angiogenesis, were shown to increase the risk of AMD [55]. Interestingly, there may also be a role for maternally inherited mitochondrial DNA (mtDNA) specifically the genes encoding for the various subunits involved in oxidative phosphorylation. Inherited variants located in the mtDNA *T2* haplogroup, characterized by 2 variants in the complex *I* gene, have also been associated with advanced AMD [56]. In addition, other variants associated with mitochondrial haplogroup *J*, *T* and *U* have also been associated with AMD [57,58].

A genetic condition referred as Stargardt disease is caused by a mutation in the ABCA4 gene also recapitulates the symptoms of macular degeneration but presents with much earlier onset, resulting in severe visual impairment and loss of central vision before the age of 20 [59]. Stargardt disease points to a significant genetic component that likely plays a role in development of AMD given that patients may progress later in life depending on variable environmental factors [3,39,59-61].

Aside from genetic factors, studies have shown that environmental and epigenetic factors also play an important role in the etiology of AMD. Gene expression during ocular development appears to be greatly impacted by the epigenetics, with respect to cell types in both the lens and retina, thus having implications ranging from early stages of disease to propensity for neovascularization during progression [62]. Concordance studies with monozygotic twins have found that nutritional and behavioral factors that influence epigenetics, such as vitamin D intake and smoking history, confer greater likelihood of developing AMD [63]. These environmental factors have been shown to significantly alter epigenetic regulation, such as methylation and acetylation, and therefore may confer a variable gene expression profile despite identical genetic information. Most recently, a study by Wei et al. showed that hypomethylation of *IL17RC* increases levels of circulating gene products, mainly inflammatory chemokines and cytokines, implicating both epigenetics and certain immune mediators in the pathogenesis of AMD [64] . Furthermore, a recent study showed that Glutathione S-transferase isoforms mu1 (*GSTM1*) and mu5 (*GSTM5*) undergo epigenetic repression in AMD RPE/choroid, which may increase susceptibility to oxidative stress in the retinas of AMD donors [65]. Another study showed that epigenetic factors regulate clusterin/*APOJ* expression, one of the proteins in drusen [65,66]. This continues to be an area of exploration, as the subject of epigenetics in AMD was recently thoroughly reviewed [67] and the field will undoubtedly continue to expand.

## AMD disease modeling

Given the complex dynamics of AMD, there have been considerable challenges in the development of an animal model that accurately recapitulates many of the characteristics of human AMD. This is, at least in part due to human genetic polymorphisms [68] and long-term exposure to environmental factors [69] that induce epigenetic changes.

In addition, human RPE cells have specific properties that are not found in currently available cell lines such as ARPE19. Human RPE cells have been generated from embryonic stem cells (ESCs) and iPS cells offering new promise for cell replacement therapy in AMD [13,15,18,70]. Stem cell biology may offer a breakthrough method for creating disease models that demonstrate the pathology of AMD in detail. Understanding the development and progression of AMD will likely offer new insight for development of potential therapies. In addition, a recent study showed that adult human RPE might contain a subpopulation of cells that are capable of self-renewal and can produce mesenchymal derivatives [71]. This observation could open new avenues for treatment of retinal degeneration by activating the dormant stem cells in the RPE.

## Current procedures & ramifications

Current treatment options in AMD can only hope to slow the progression of disease, although a recent review of the literature suggests that the field of AMD therapy is dynamically changing and growing rapidly, with some strategies seeking to correct the damage of AMD [72]. Most therapies that are currently utilized in the clinic have shown mild success in slowing degeneration of RPE and preventing the onset of neovascularization. Laser therapy has been shown to significantly reduce drusen accumulation in patients with dry AMD within a three-month period post-operation [73]. However despite the overall reduction in drusen with this laser photocoagulation, the risk of later developing choroidal neovascularization (CNV), geographic atrophy, or loss of central vision is not reduced [74]. In fact, studies have shown that patients given higher intensity laser therapy are at a higher risk of developing choroidal neovascularization [75].

Anti-angiogenic therapies are currently FDA-approved for neovascular AMD, with clinical trials showing significant improvement in visual acuity and slowed progression of disease [76]. It has been shown that patients with neovascularization demonstrate abnormally high levels of VEGF-A in the choroidal layer and vitreous humor and that this expression contributes greatly to the growth and proliferation of immature capillaries [77,78]. These vessels demonstrate abnormal capillary lumens and increased permeability, making them particularly susceptible to spontaneous hemorrhage, thereby causing significant macular damage [77,78] . The anti-VEGF treatment helps to decrease the formation of new vessels and prevent further infiltration of the choroidal layer into the nearby RPE. Numerous studies have shown clinical efficacy for ranibizumab and bevacizumab, monoclonal antibodies that specifically bind VEGF-A [79,80]. Both antibodies have demonstrated efficacy in slowing vision loss and improving visual acuity [81,82]. However, some serious side effects have been noted including macular hemorrhages and retinal detachment [83].

A surgical technique has also been designed for treatment of AMD involving the partial or total translocation of the macula to area of less diseased RPE [84,85]. This approach has resulted in improved visual acuity for a percentage of patients, however it presents significant complications, including fibrosis and widespread failure of RPE survival on Bruch’s membrane despite minimal improvements in vision, bleeding, corneal astigmatism, and retinal detachment with proliferative vitreoretinopathy [86-88]. Many patients also experience tilting of the visual image or diplopia after retinal rotation [84]. Given the complications associated with the surgical procedures, retinal translocation efforts have been limited. However, the concept of utilizing a healthy RPE layer persists and has inspired the implantation of non-diseased RPE cells derived from donors and stem cell-based therapies for replacement of the disease cells in the retina.

## Cellular transplant as therapy for AMD

AMD is initiated with the dysfunction and death of RPE, leading to photoreceptor loss and significant deficits in vision. Therefore, the key in successful cell-based therapy in AMD would be early replacement of the damaged RPE [21]. Several studies have shown that transplanted RPE cells have the potential to rescue photoreceptors [89-91]. To date, a number of studies have investigated various stem cell types as potential sources for retinal transplantation including ESCs, adult stem/progenitor cells and more recently induced pluripotent stem cells (iPSCs) [92-94]. Use of stem cells for retinal repair offers enormous promise for generation of adequate and appropriate cell populations for transplantation. Subretinally transplanted RPE that were differentiated from ESCs have led to improvements in visual acuity in preclinical models of the disease [16,70] In addition, human iPSCs have been differentiated towards functional RPE cells, and we have demonstrated that human iPSC-derived RPE are functionally and phenotypically similar to native RPE [18]. Unfortunately, the subretinal transplantation of RPE cell suspensions in the Royal College of Surgeons (RCS) rat model, a genetic model of RPE degeneration, has only resulted in short-term survival and maintenance of photoreceptors [14]. Therefore, the efficiency of cell delivery and the degree of visual rescue often remain unsatisfactory, despite the apparently positive findings [95-97]. This lack of efficacy may be due to a number of reasons: 1) RPE cells are adherent monolayer cells and therefore must attach to a compliant matrix following transplantation, 2) the basal lamina layer of Bruch’s membrane may be damaged or absent in advanced retinal disease, with age, or following macular surgery [98], lacking the supportive structure upon which RPE cells are normally attached; thus, it is difficult for newly transplanted cells to attach in such a non-tolerant environment, 3) transplanted cells may clump together rather than forming appropriately polarized monolayer RPE [99]. Furthermore, lack of cell-to-cell contact may also lead to transition of RPE cells to inappropriate phenotypes such as epithelial-mesenchymal transition [97,100].

Therefore, the gap between theory and clinical exploitation remains considerable [101,102]. In addition, safe and efficient tissue delivery needs to be considered, as do survival and integration of the transplanted cells within the host [103-105]. Any transplanted material must also be capable of maintaining an appropriate state of differentiation. In addition, immune surveillance is a significant issue, and so the approach of autologous sources of cells for transplantation to negate problems with graft rejection would be ideal [106].

## Biomaterials and cell delivery scaffolds

It has been documented that cells injected as a suspension often fail to survive and to regain a fully differentiated phenotype [90,107]. In addition, the viability of RPE cells delivered to the subretinal space is often dependent on the integrity of the underlying substrate, the Bruch’s membrane [108,109]. Thus, transplantation of a polarized RPE monolayer as a sheet seems to be more promising. Studies have shown that scaffolds made of biodegradable polyester such as poly (L-lactic acid) (PLLA) and poly (D, L-lactic-co-glycolic acid) (PLGA) could improve cell survival and organization of retinal progenitor cells (RPCs) and promote differentiation of the RPCs towards mature retinal cell phenotypes [110]. These polymers were selected, as they are biocompatible, relatively easy to process and have been successfully used for tissue engineering applications [111,112]. The degradation rate of these polymers can also be manipulated by changing properties such as molecular weight and the ratio of lactic to glycolic units. Thus, polymers can be designed to degrade over the most appropriate timescale for the desired application. Several other polymers and preparation techniques have also been investigated. Many factors such as surface chemistry, mechanical properties and surface topology can affect the practicability of different materials for cell attachment and survival. Examples of other polymers are: Poly (methyl methacrylate) (PMMA) that has been used to manufacture ultrathin, micro-machined scaffolds for RPCs [113]. Similarly, poly (glycerol sebacate) (PGS) [114-116] has been used to manufacture a porous, elastic scaffold and poly (ε-caprolactone) (PCL) [117] to produce ultrathin nanowire scaffolds. These polymers have supported successful growth of murine retinal progenitor cells both *in vitro* and *in vivo* in degenerative mouse models.

## Stem cells in AMD

### Human embryonic stem cells

Human embryonic stem (hES) cells have dramatically altered the field of cellular biology since the first lines were established in 1998 [10]. With regard to retinopathies like AMD and Stargardt disease, these cells have shown commitment to RPE formation *in vitro* in response to culturing techniques that direct differentiation towards the RPE lineage [16,118-121]. Furthermore, *in vivo* subretinal transplant of purified hESC-derived RPEs into the Royal College of Science (RCS) rat and the Elov14 mouse, an animal model for Stargardt disease, have shown marked improvements in visual function [118] and survival of the graft without teratoma formation or cellular hyperproliferation [14,16].

Although these data are promising, the use of hESCs is challenging due to the ethical issues, the immunological reaction and the long-term risks of teratoma formation [10]. The field continues to improve culturing techniques for hESC-RPEs by reducing the need for co-culture or animal growth factors [122]. HESCs express human leukocyte markers (HLA) that mediate immune responses, thus making hESC-RPE grafts susceptible to rejection response by the recipient, despite relative immune-privilege in the subretinal space. Therefore therapies using hESCs require administration of immunosuppressive drugs that may induce complications in elderly patients.

The limited available number of hESC cell lines tends to limit the quality of these cultured cells, particularly with extended culture and expansion, which results in a decline of surface receptors, enzymatic activity and overall loss of cellular polarization in hESC-RPE differentiated lines [123]. Efforts to develop refined culturing technique, and methods for the necessary large-scale expansion of these delicate cell populations must be explored before hESC therapy can become a reality for patients with AMD. However the ethical concerns will always limit the use of ESCs at least in certain countries.

### Induced pluripotent stem (iPS) cells

Recent studies have shown that the donor cell type can influence the epigenome and differentiation potential of iPSCs [124-132]. For example, non-hematopoetic iPCs will show a less robust differentiation towards blood cells than those that were originally of hematopoetic origin and vice versa, likely due to repressive methylation patterns that persist at loci necessary for commitment to that particular lineage [127]. Moreover, it has been shown that human iPSCs derived from RPE (RPE-derived iPSCs) retain the epigenetic memory of their tissue of origin (RPE) [133]. Understanding the dynamics and implications of this biology is necessary prior to implementation of iPSC therapies in human subjects to accurately predict outcome and reduce risk.

## Future directions of stem cell therapy in AMD

Perhaps among the most promising clinical stem cell study to date is a very small Phase I clinical trial in treatment of AMD with transplant of hESC-RPEs (NCT01344993). These patients received a cellular suspension graft of >99% purified hESC-RPEs injected into the subretinal space. Four months following transplant, neither patient showed formation of teratoma or tumor at the site of injection and both reported improved visual acuity, although placebo effect has not been assessed. This trial utilized minimum cell numbers in transplant which has been shown to reduce likelihood of teratoma formation and also secured an extraordinarily high level of hESC-RPE purity, thus reducing the possibility of aberrant differentiation of cells that had retained pluripotency. Results from this small clinical experiment are to be met with conservative enthusiasm, given the very limited sample size and modest benefit. At this time, the trial will be expanded to a greater cohort of patients, with the results anticipated in January/June 2013. This open-label Phase I/II trial seeks to determine the safety and tolerability of this procedure and represents one of the first clinical trials involving the use of stem cell transplant in treatment of macular dystrophy and related retinopathies [134].

RPE transplantation for the neuroprotection of retinal photoreceptors within the retina in AMD is among the first application of hESC transplant clinically. Restoration of an intact RPE layer will likely prevent progression and further deterioration of the photoreceptors, improving the microenvironment needed for survival of the remaining retinal cells. HESCs and iPSCs have successfully been differentiated into functional RPE and photoreceptors *in vitro*[15,18,121,135]. Application of these cultures may be successful in the future after development of more advanced techniques in transplant and scaffolding. *In vivo* models of photoreceptor dysfunction, particularly the Crx-deficient (*cone-rox homeobox)* mouse model, responds to photoreceptor transplant with integration of hESC-photoreceptors into the subretinal space and increased responses to light stimuli [135]. Photoreceptor transplant in macular degeneration may also confer a great therapeutic avenue in the future for rescue visual acuity.

## Conclusions

The wealth of data from stem cell-derived RPE in disease-models and clinical trials will undoubtedly yield important insight in understanding the mechanisms of AMD and developing effective treatment strategies. The iPS-derived RPE opens new avenues for generation of a “disease in a dish” model of AMD that otherwise would not be possible to recreate. For cell transplantations, concerns remain regarding the process of pluripotency induction and residual epigenetics in iPS cells, particularly since there are unique characteristics that may significantly affect the propensity of differentiation and may influence ongoing attempts to use iPSCs for disease modeling. Human embryonic stem cells also present dangers associated with teratoma formation, which must be understood and controlled prior to implementation of successful wide-scale clinical trial. Consequently, identifying and understanding the markers of disease and therapeutic response by generating an *in vitro* disease model of AMD will undoubtedly yield more innovative therapeutics that will target the etiology of AMD. Given the mild success of RPE transplant, at this time, efforts to maximize RPE survival and integration at the subretinal region are paramount for success of this therapeutic strategy.

## Abbreviations

AMD: Age-related Macular Degeneration; RPE: Retinal-Pigmented Epithelium; IPS: Induced Pluripotent Stem cell; VEGF: Vascular Endothelial Growth Factor; ESC: Embryonic Stem Cell; hECS: human Embryonic Stem cell; CFH: Complement Factor H; C2: Complement 2; CFB: Complement Factor B; C3: Complement 3; CFHR1: Complement Factor H-Related gene; CFI: Complement Factor I; ARMS2: Age-related Maculopathy Susceptibility 2; APOE: Apolipoprotein E; CETP: Cholesteryl Ester Transfer Protein; GSTM1: GSTM5, Glutathione S-transferase isoforms mu1, mu5; PLLA: Poly (L-Lactic) Acid; PLGA: Poly D, L-Lactic Co-Glycolic Acid; RPC: Retinal Progenitor Cells; PGS: Poly Glycerol Sebacate; PCL: Poly Ε-Caprolactone.

## Competing interests

The authors declare that they have no competing interests.

## Authors’ contributions

HM: Wrote, edited, prepared the manuscript for publication. MC: Wrote, edited, prepared the manuscript for publication. KH: Wrote and edited the manuscript. AS: Wrote and edited the manuscript. NG: Wrote, critically revised and added additional intellectual content to the manuscript. All authors read and approved the final manuscript.
